# Evidence-Based Self-Management Strategies for Fibromyalgia: Foundations for Digital Therapeutic Applications

**DOI:** 10.2196/67523

**Published:** 2026-02-17

**Authors:** Thomas Lebeau Foustoukos, Isabelle Leclercq, Marc Blanchard, Thomas Hügle

**Affiliations:** 1 College of Medicine and Veterinary Medicine University of Edinburgh Edinburgh United Kingdom; 2 Department of Rheumatology University Hospital of Lausanne Lausanne, Vaud Switzerland

**Keywords:** chronic pain, cognitive behavior therapy, digital therapeutics, eHealth, fibromyalgia, mind-body therapies, mobile application, physical activity, self-management

## Abstract

Fibromyalgia is a prevalent musculoskeletal pain condition that causes major personal, social, and societal burden. Pharmacological therapies often provide only limited benefit, making multimodal approaches and self-management the cornerstones of care. Such strategies, spanning lifestyle modification, physical activity, psychoeducation, and cognitive-behavioral approaches, target the biopsychosocial complexity of fibromyalgia and promote sustainable coping. In parallel, digital health technologies are transforming how these interventions can be delivered and coordinated in the form of digital therapeutics. This viewpoint draws on a multiphase investigation to appraise the current and future landscape of fibromyalgia self-management in the digital era. Its objective is to present an evidence-based framework and recommendations to guide the development of a mobile health self-management program for patients with fibromyalgia. In phase 1, we conducted a review of international guidelines and randomized controlled trial–based systematic reviews addressing nondigital self-management interventions for fibromyalgia and related nociplastic pain conditions. In phase 2, we analyzed the content and certification status of currently available mobile and virtual health applications for fibromyalgia. In phase 3, we convened a multidisciplinary focus group of rheumatologists, patients, and digital health developers to identify priorities for translating evidence-based self-management content into mobile health formats. Collectively, we suggest that effective digital self-management for fibromyalgia should evolve beyond single-domain interventions toward validated, personalized, and interactive multimodal platforms. Virtual care may increasingly function at the point of care, linking monitoring, education, and behavioral support in one continuum.

## Introduction

Fibromyalgia is defined as a syndrome characterized by moderate to severe symptoms, including widespread pain, fatigue, sleep disturbance, cognitive complaints, and an increase in somatic complaints [1]. Despite ongoing efforts to clarify the definition of fibromyalgia, accurately assessing its diagnosis, prevalence, management, and its personal and societal impacts remains challenging [2]. Fibromyalgia can be accompanied by psychiatric, rheumatological, gastrointestinal, or other comorbidities, which further complicate the management and contribute to its overall personal and societal burden [3].

Proposing a clear treatment strategy for fibromyalgia remains challenging. The focus is on nonpharmacological therapies, particularly physical interventions, psychological treatments, and mind-body approaches that aim to promote self-management. Patient education plays a crucial role in enhancing their understanding of the disease and fostering their active participation in its management. Pharmacological treatments have shown limited benefits [4-8]. Multimodal treatment programs are recommended for treatment-resistant fibromyalgia patients, as they combine different treatment modalities and offer group effects. However, such programs require significant resources that are not necessarily considered cost-effective.

Self-management programs play a crucial role in supporting individuals with chronic conditions [9] and are recommended by management fibromyalgia guidelines [4-7]. The fundamental concept of self-management emphasizes the active involvement of patients [9,10]. Self-management considers the multidimensional nature of the illness and combines multiple interventions such as psychological interventions, exercise, and nutritional aspects. It offers a relevant approach to address the challenges in fibromyalgia management and shows its efficiency to improve fibromyalgia patients’ well-being [11,12] and a reduced tendency of patients to seek repeated consultations with health care professionals. A plethora of these programs have shown an improvement in pain intensity, functionality, cognitive behavior, emotions, and quality of life (QoL). However, the quality of these studies is heterogeneous [13-17]. When programs are detailed, their references are often unclear, relying on peer opinions or interviews [18-24], or they may offer only one component, such as psychoeducation or exercises [25-28].

The digital transformation is continuously shaping the health care landscape. It encompasses various digital innovations such as online programs, apps, gaming, virtual reality, or, more recently, generative artificial intelligence (AI) and chatbots [29]. In a clinical context, mobile health (mHealth) apps have demonstrated several key functionalities, including diagnostics and clinical decision support, behavior change interventions, and the delivery of disease-related education and communication support. The latter 3 functionalities are particularly relevant for delivering self-management interventions, empowering patients to monitor and manage their own health conditions, and providing an additional avenue for health care professionals to support their patients’ well-being [30]. In 2019, a law was passed in Germany that digital health applications are reimbursable by health insurance companies under certain conditions. Currently, 8 digital health applications for musculoskeletal indications are registered; 2 of those are for chronic pain syndromes, including fibromyalgia [31]. There was a second acceleration of eHealth during the COVID-19 pandemic, notably concerning telemonitoring and video consultations. In the meantime, eHealth interventions have been shown to enhance patients’ QoL and treatment experiences [32,33].

Online programs and mobile apps have demonstrated promising benefits for individuals with chronic pain or musculoskeletal conditions [33,34]. The use of mHealth technologies offers an accessible and available 24/7 solution without geographical constraints, empowering patients to become more self-reliant in managing their chronic conditions [35,36]. Notwithstanding, there are several caveats and limitations in existing solutions. A substantial proportion of currently available online fibromyalgia programs are not evidence-based or lack proven, evidence-based content. They are usually not personalized and cannot be updated, as this would alter the certification status of the device. On the other hand, recent digital tools such as large language models (LLMs) and chatbots offer novel approaches that could address some of these limitations.

## Aim

The objective of this work is to present an evidence-based framework and recommendations to guide the development of an mHealth self-management program for patients with fibromyalgia. A comprehensive literature review was conducted to evaluate the effectiveness of self-management interventions on symptoms in patients with fibromyalgia (phase 1). Where applicable, relevant practical characteristics of the interventions were also reported. In phase 2, existing therapeutic fibromyalgia apps and online programs were reviewed, and in phase 3, general aspects and user preferences were discussed in focus groups with patients. Finally, we aimed to identify strategies to optimize online self-management interventions and explore their potential for further improvement.

## Ethical Considerations

This study is based on previously conducted studies and does not contain any new studies with human participants or animals performed by any of the authors.

## Phase 1: Review of Existing Evidence

### Overview

MEDLINE and Cochrane databases, encompassing English and French articles from the inception of the databases up until 2023, were searched to identify guidelines and systematic reviews of randomized controlled trials for fibromyalgia self-management. Data from 30 systematic reviews that demonstrated a high or moderate level of confidence based on the AMSTAR 2 evaluation and 8 guidelines with an AGREE II–GRS score higher than 18 were considered to categorize and summarize the evidence on self-management interventions (Figure S1 in Multimedia Appendix 1).

### Categorization of Self-Management Interventions

Self-management interventions were categorized into 3 groups: physical exercise, psychoeducative interventions, and mind-body interventions. Additionally, a separate category called multimodal interventions was identified, which encompassed interventions combining 2 or more of the previously mentioned self-management approaches. The European, American, Canadian, and Italian guidelines covered physical exercise, psychoeducative interventions, and mind-body self-management interventions as part of the overall management of patients with fibromyalgia, alongside other treatment modalities [4,6,7,37]. The German guideline divided its recommendations into 4 separate studies: general recommendations [7], recommendations on physical interventions [38], psychological interventions [39], and other interventions such as mind-body therapies [40]. In total, 8 studies focused on physical activities [41-48]; 14 studies on psychoeducative interventions [25,49-60]; 4 studies on mind-body therapies [61-64]; 1 study on multicomponent interventions [11]; 1 study that investigated both psychoeducative and mind-body interventions [65]; and 2 studies that examined physical activities, psychoeducative interventions, mind-body therapies, and multicomponent interventions [66,67]. The studies used various types of control groups, which encompassed the following options: treatment as usual, waiting list or no treatment, alternative pharmacological or nonpharmacological interventions, active or attention control, and placebo or sham interventions. Designing a control group for complex self-management interventions was considered challenging due to the inherent difficulty of blinding participants, which can introduce measurement and interpretation bias [68].

### Evidence for Specific Self-Management Interventions

A wide range of variables were assessed both before and after the interventions: pain (both qualitative and quantitative measures), QoL, psychological functions such as depression, anxiety, self-efficacy, acceptance, catastrophizing, and fear-avoidance, physical functions such as strength and disability, sleep quality, fatigue, health care use indicators such as visits, sick leaves, return to work, and care-seeking behavior, as well as trial withdrawal rates and adverse effects. The Fibromyalgia Impact Questionnaire (FIQ) was used as a multidimensional measurement tool.

### Physical Interventions

All of the guidelines strongly endorse the inclusion of aerobic exercises and resistance (or strength) exercises in self-management interventions for fibromyalgia [4,6,7,37,38]. On the other hand, flexibility and stretching exercises are either not recommended [6,7] or recommended with weak or insufficient evidence of benefit [37,38]. Exercise interventions were found to be superior to control groups in improving pain, FIQ scores, sleep quality, fatigue, and depression [67]. The characteristics of the exercises were extracted based on the prescription guidelines provided by the American College of Sports Medicine, which include frequency, intensity, time, and type of exercises [69]. Additionally, the duration of the exercises, delivery modalities, whether they were tailored or standardized, and safety considerations were also considered. Aerobic exercises had positive effects on pain, QoL, and depression [42,45,46]. Aerobic exercises demonstrated a high effect on reducing the FIQ [41]. Aerobic exercises probably improve stiffness and slightly improve physical function and cardiovascular function [42]. According to Couto et al [46], resistance exercises yielded positive effects on pain and QoL but did not show a significant effect on depression. On the other hand, Albuquerque et al [41] found that resistance exercise demonstrated a moderate effect in reducing the FIQ scores. Resistance exercise has the potential to improve fatigue and sleep [49]. Although flexibility exercise did not show superiority over control groups (other interventions or no intervention), the quality of evidence is insufficient to draw definitive conclusions regarding the effectiveness of flexibility exercise on fibromyalgia symptoms [41,45,46,48]. However, there is evidence suggesting that physical exercise may help decrease muscle stiffness [48]. Interventions that combine aerobic exercises, resistance exercises, and flexibility exercises have shown greater effectiveness in improving FIQ scores compared to single-type exercise interventions [41]. However, Bidonde et al [43] state that no definitive conclusion can be drawn regarding the optimal proportion, synergy, or specific characteristics of each exercise type [43]. Exergames have demonstrated promising benefits in improving pain, disability, and physical function in both the short and long term [66]. There is a lack of available information regarding other exercises, such as Pilates and motor control exercises. Exercise (aerobic, resistance, and flexibility) characteristics are shown in Table S1 in Multimedia Appendix 1.

### Psychoeducative Interventions

Consensus guidelines recommend the inclusion of psychological and educational interventions, such as cognitive behavioral therapy (CBT) and health education [4-7,37]. These interventions can encourage self-management, enhance self-efficacy, and reduce maladaptive thoughts and behaviors [6]. It is particularly important to consider these interventions when patients have comorbid mental disorders [39]. For hypnosis and guided imagery, recommendations are conflicting [4,7,39]. Although other psychological interventions, such as relaxation, therapeutic writing, Roger therapy, family therapy, psychodynamic therapy, and psychoanalytic therapy, are available, they should not be proposed as stand-alone treatments due to the lack of evidence or limited recommendations [39]. CBT incorporates education on the physiopathology of pain, self-management skills, cognitive reappraisal, pacing activities, and problem-solving techniques to help patients modify their behaviors, thoughts, and emotions, ultimately reducing pain, improving functioning, and enhancing mood. Additional components, such as sleep hygiene, may be included. Homework assignments are given to encourage the practice of skills in everyday life [60]. Acceptance and commitment therapy (ACT) and mindfulness-based therapy are extensions of CBT. ACT comprises 2 main core components: mindfulness and acceptance, as well as commitment and behavior change. It involves several processes, including acceptance, cognitive defusion, present-moment awareness, self-as-context, values clarification, and committed action [70]. ACT interventions often incorporate CBT processes, making it difficult to distinguish between CBT and ACT [51]. Evidence suggests that CBT can reduce pain, disability, and negative mood in patients with chronic pain or fibromyalgia, both immediately after treatment and at long-term follow-up (6 and 12 months), although the effect size is small or very small [68,79. Enomoto et al [52] recommend offering CBT for insomnia, or at least CBT for insomnia and pain, to patients with chronic pain who also experience insomnia as a comorbidity. CBT therapy for insomnia has been effective in improving sleep, pain, disability, and depression. ACT has also demonstrated benefits for patients with chronic pain, improving pain acceptance, QoL, pain intensity, functioning, and mood [57,59]. For fibromyalgia, ACT has shown significant improvements in patient functioning in both the short and long term, based on a meta-analysis with moderate-quality evidence [51]. ACT can be considered to enhance patients’ psychological flexibility and, subsequently, their functioning in pursuing valued activities [51,59]. Education serves as both an intervention and a CBT tool [58]. Joypaul et al [55] defined education as providing instructions to inform participants, making it applicable to various interventions as an instructional tool [55]. Therapeutic pain neuroscience education, also referred to as pain neuroscience education by some authors, aims to enhance patients’ knowledge and understanding of pain neurophysiology to improve their cognitive and behavioral skills related to pain [56,71]. Therapeutic pain neuroscience education has demonstrated benefits for patients with chronic pain in reducing fear of movement, pain intensity, pain disability, and pain catastrophizing [58]. Therapeutic pain neuroscience education, in the context of chronic musculoskeletal pain, improved pain intensity, pain knowledge, disability, maladaptive thoughts and behaviors, physical function, and health care use, even up to 1 year post treatment [56]. Other educational content has been studied, including lifestyle components focusing on areas such as nutrition, sexuality, social coping strategies, and the regulation and adjustment of everyday life [11]. Education should be an integral part of a multidisciplinary approach to chronic pain, alongside graded physical activities, graded exposure, and pacing, for example, and not offered as a stand-alone treatment [54-56,58,67]. CBT education characteristics were gathered in Table S1 in Multimedia Appendix 1.

### Mind-Body Interventions

The classification of mind-body interventions was based on the Medical Subject Headings terms of the National Library of Medicine. They encompass various modalities such as meditative movement therapies (MMTs), respiration exercises, hypnosis and autogenic training, meditation, and relaxation [72]. They focus on exploring the interconnectedness between the brain, body, mind, and behavior, and how emotional, mental, social, spiritual, experiential, and behavioral factors can directly impact health [73]. Guidelines recommend the use of MMTs with confidence [4,7,40]. However, hypnosis, guided imagery, relaxation, or meditation are not recommended, and it is advised not to propose them as stand-alone interventions [7,40]. The Canadian guideline suggests not discontinuing these interventions but informing the patient about their lack of evidence and potential side effects [6]. Therapies such as Qi Gong, Tai Chi, and Yoga, as examples of MMTs, were included [63]. MMTs have shown improvements in outcomes such as physical functioning, pain, and mood for patients with fibromyalgia [65]. Specifically, Tai Chi has been found to significantly reduce FIQ scores, pain intensity, sleep disturbance, fatigue, and depression, while increasing QoL for individuals with fibromyalgia [61]. Tai Chi and Yoga are weakly recommended, whereas the evidence did not allow for a recommendation regarding Qi Gong for chronic pain management [63]. Lee et al [64] did not provide a recommendation for meditation as a treatment for chronic pain due to the limited evidence available. While no specific recommendation was provided in the systematic review, Lee et al [64] suggested that mindfulness-based interventions could potentially offer benefits for chronic pain management.

### Multicomponent Interventions

Multicomponent interventions were defined as combinations of at least 2 components, including psychological, physical activity, medical education, and mind-body therapies, based on the self-management program developed by Miles et al [74] and further refined by Geraghty et al [11]. All guidelines recommend not implementing interventions in isolation but rather combining them with other approaches. Multicomponent intervention should include physical and psychoeducational interventions at a minimum [4-7,75]. Multicomponent interventions have demonstrated effectiveness in improving various aspects such as FIQ, pain, sleep, and depression, with greater effects compared to exercise alone, education alone, or psychological interventions alone [61,62]. The positive effects of multicomponent interventions typically last for an average of 14 weeks, and it is recommended to conduct follow-up assessments every 3 months to review and reinforce the treatment strategy [62]. Considering the variations across studies, multicomponent interventions have shown improvements in physical function, pain, FIQ, fatigue, mood, and QoL in both the short and long term [11].

## Phase 2: Review of Self-Management Apps

In this part of the work, we investigated which type of self-management is integrated in existing apps for fibromyalgia and chronic pain syndromes (Table 1). Online fibromyalgia applications were identified via PubMed, Google, and the German Digitale Gesundheitsanwendung (DiGA) registry [76], as well as with ChatGPT (OpenAI). Available mobile apps targeting fibromyalgia self-management reveal a strong emphasis on psychoeducational content, particularly CBT, ACT, and pain neuroscience education. Out of the 12 reviewed apps, 10 integrate psychoeducational modules, often presented through structured lessons, interactive exercises, journaling tools, or virtual coaching. Physical activity interventions, such as aerobic or resistance exercise guidance, were integrated in 6 apps, typically via instructional videos, activity logging, or synchronization with wearable devices. Mind-body techniques, including guided meditation, breathing exercises, and mindfulness-based practices, were found in about 5 apps. This distribution highlights a predominant focus on psychological and cognitive strategies in current digital interventions, with fewer apps addressing physical or somatic components in depth.

**Table 1:** Existing online self-management programs for fibromyalgia.

App name	Therapeutic mechanisms	Regulatory status	Notes
Stanza	ACT^a^, CBT^b^, mindfulness, journaling, reminders	FDA^c^-cleared in the United States	12-week structured digital therapy, clinical trial support
HelloBetter Chronic Pain	CBT, ACT, mindfulness	DiGA^d^-approved in Germany	Structured 12-week course, reimbursed by German health insurance
Selfapy Chronic Pain	CBT, mindfulness, education	DiGA approval in Germany withdrawn	Evidence-based course, reimbursed via DiGA
Manage My Pain	Pain tracking, journaling, self-monitoring	Available on app stores	Focuses on tracking pain trends for self-awareness and provider communication
Quell Fibromyalgia	Neuromodulation via wearable, symptom tracking	FDA-authorized medical device	App supports wearable neuromodulation therapy for fibromyalgia
FibroMapp	CBT-based tools, symptom tracking, medication management	Available on app stores	Combines tracking and CBT tools; user-driven format
MoreGoodDays	CBT, mindfulness, education	Available on app stores	Structured CBT modules, lifestyle education, mindfulness tools
FibroMinder	Reminders, symptom tracking, task management	Available on app stores	Simple utility app for scheduling and symptom tracking
PainScale	Pain tracking, education, CBT elements, community support	Available on app stores	Community-focused platform with integrated education and logging
FibroTrack	Symptom tracking, lifestyle management	Available on app stores	Focuses on tracking and lifestyle planning features
Curable	Pain neuroscience education, CBT, guided meditation, writing exercises	Available on app stores	Comprehensive app targeting pain beliefs and coping strategies
Fibrowalk	CBT, mindfulness, therapeutic exercise, pain neuroscience education	Not a certified app; delivered via YouTube and email	Multicomponent program with weekly video sessions

^a^ACT: acceptance and commitment therapy.

^b^CBT: cognitive behavioral therapy.

^c^FDA: US Food and Drug Administration.

^d^DiGA: Digitale Gesundheitsanwendung.

Two applications, HelloBetter Chronic Pain and Stanza, are certified digital therapeutics. HelloBetter is DiGA-approved in Germany, meaning they are reimbursable by statutory health insurance and have met criteria for clinical evidence and data security. Stanza, developed in the United States, is US Food and Drug Administration–cleared as a prescription digital therapeutic, offering a 12-week ACT-based program with demonstrated clinical efficacy in reducing fibromyalgia symptoms [77]. The remaining apps, including Curable, MoreGoodDays, Manage My Pain, and FibroMapp, are publicly available but lack medical certification and are typically framed as wellness tools or digital companions rather than regulated treatments. Overall, while several apps offer well-designed psychoeducational modules and basic symptom tracking, fewer integrate evidence-based physical activity interventions or comprehensive mind-body components. Among the 12 fibromyalgia self-management apps analyzed, only 5 (42%) can be considered multicomponent, meaning they integrate at least 2 of the 3 core evidence-based intervention domains: psychoeducational therapies, physical activity, and mind-body techniques. Notably, the certified apps HelloBetter Chronic Pain, Selfapy Chronic Pain, and Stanza fall into this category, offering combinations of CBT or ACT, mindfulness, and, in some cases, activity planning. Noncertified apps such as MoreGoodDays and Curable also include psychoeducation and mind-body interventions, but vary in the depth and clinical rigor of their content. While most of the reviewed apps integrate evidence-based components aligned with current clinical guidelines, such as CBT, ACT, and mindfulness-based techniques, several also include strategies with limited or inconclusive evidence in fibromyalgia. For example, some noncertified apps incorporate generic meditation, unstructured journaling, expressive writing, or broad lifestyle advice without therapeutic framing or individual tailoring.

## Phase 3: Patient Preferences and Focus Groups

As previously reported, we investigated patient preferences for mobile app design through 2 online surveys (53 and 33 patients, respectively) and 3 focus groups comprising a rheumatologist, 1-6 fibromyalgia patients, with or without concomitant post-COVID-19 syndrome, as well as an app designer [78]. All patients in focus groups tested a self-developed app designed to assess fibromyalgia symptoms and to deliver an online therapeutic program containing CBT, mind-body techniques, and physical exercise instructions. This program was initially developed during the pandemic for fibromyalgia associated with postviral syndrome and was subsequently adapted as an experimental chronic pain companion (Pain Organiser and Companion System). The design and content of this app are described in detail elsewhere [78,79]. In the final focus group (n=1), the online program was connected to an LLM-based chatbot and tested by the patient (see Future Directions section).

Participants emphasized that simplicity, clarity, and accessibility are crucial for adherence, given common cognitive fatigue (“fibrofog”) and limited digital literacy. Users preferred short, well-structured modules, clear navigation, and scientifically validated content in plain, nontechnical language. Autonomy and empowerment emerged as central themes. Patients valued tools that visualize progress, such as symptom tracking and diaries, helping them recognize links between behavior and symptoms. Personalization was highly desired; users wanted adaptable exercises, favorite lists, and an interactive virtual coach or chatbot that responds to individual needs and previous activity. An empathic, motivating tone was considered essential. Participants appreciated friendly communication and multimedia content, particularly physiotherapy videos and mindfulness modules. Emotional design elements, positive wording, supportive colors, and encouraging feedback were viewed as vital for engagement and adherence. Reliability and trustworthiness were further priorities: users expected apps to be evidence-based, transparent, and secure, with clear data protection and professional validation. Barriers such as poor navigation, cognitive overload, or lack of emotional connection can significantly reduce adherence. The focus group highlighted that fibromyalgia mHealth apps offer the potential to serve as multicomponent platforms, covering the 3 key therapeutic domains discussed above: psychoeducation, mind-body techniques, and physical exercise guidance. Of note, instructions for physical exercises were appreciated by participants in the focus groups, ideally with more individualization (eg, for individuals with obesity).

## Key Findings and Lessons Learned

There is proven evidence for psychoeducational interventions, mind-body therapies, and physical activities in fibromyalgia. All 3 are included in most of the current fibromyalgia mHealth applications on the market, several of those with proven efficacy in randomized controlled trials, although the underlying therapeutic content is not openly accessible [75]. CBT remained strongly recommended to assist patients with fibromyalgia in managing their pain, comorbidities, and lifestyle [49] and is the main approach used in existing fibromyalgia apps. Apart from CBT interventions, psychoeducation focuses on pain coping skills, mindfulness, lifestyle modification, and pain neuroscience education. The recommendations regarding mind-body therapies such as MMT, meditation, relaxation, or hypnosis are conflicting but safe and therefore might be integrated in apps as an adjunct. Certain interventions, such as stress reduction, breathing, and relaxation exercises, are notably suitable for integrating into apps as animation can be positive, such as a balloon for breathing exercises.

Half of the existing fibromyalgia apps integrate physical exercises into their program, but mostly only to a lower degree compared to CBT. The content of existing apps contrasts with technically more sophisticated physiotherapy or online rehabilitation apps; for example, integrating computer vision to instruct and monitor a wide range of exercises. In general, it can be postulated that true multicomponent apps integrating all 3 validated pillars of fibromyalgia self-management, psychoeducation, physical activity, and mind-body techniques, guided by clinical standards and codeveloped with patient input, would improve the effect.

From a technical side, both web and native apps are viable platforms for integrating self-management tools and tracking disease activity in fibromyalgia. Native apps are particularly effective at incorporating wearable data directly from mobile devices, enabling continuous monitoring and data capture. Symptom tracking is typically based on patient-reported outcomes or symptom scores, including measures of pain, QoL, depression, anxiety, self-efficacy, acceptance, catastrophizing, fear-avoidance, physical function (eg, strength and disability), sleep quality, fatigue, and health care use indicators such as clinic visits, sick leave, and return-to-work patterns.

These apps often incorporate validated instruments such as the FIQ, the Symptom Severity Score, or the Widespread Pain Index to assess disease activity. Therapeutic content is typically delivered through text, animated videos, or voice formats, providing education, instructions, and exercises. In some cases, apps are integrated with connected devices, such as wearables that deliver millimeter wave stimulation [80]. Animation can be used to enhance certain exercises, such as balloons being used to synchronize breathing frequency and intensity.

Stand-alone apps, particularly those certified as digital therapeutics, often include a structured program guide to track patient progress. This is important for potential reimbursement by health insurers. Increasingly, mHealth apps also integrate chatbots that guide users through the program, enhance flexibility, and potentially improve adherence, one of the main challenges of these interventions. Successful therapeutic apps for this population require a balance between scientific rigor, user-friendly design, and emotionally intelligent interaction. A clinical and evidence-based framework for fibromyalgia interventions in apps can be found in Table 2. Textbox 1 presents practical recommendations for the corresponding app design and implementation roadmap.

**Table 2:** Evidence-based framework for fibromyalgia online interventions.

Intervention domain	Evidence level and rationale	Implementation in mobile health app	Digital enablement features	Evaluation metrics
Psychoeducational therapies (CBT^a^, ACT^b^, and PNE^c^)	Strong evidence from RCTs^d^ and guidelines for reducing pain, distress, and maladaptive thoughts	Modular courses; microlearning sessions; quizzes; journaling; goal setting; and self-efficacy tracking	Chatbots or avatars for guidance; adaptive progression based on symptom and mood data; empathy-based conversational tone; and voice message transcription	Pain, FIQ^e^, self-efficacy, catastrophizing, engagement rate, and module completion
Physical activity and graded exercise	Strong evidence for aerobic and resistance training improving function and mood	Video demonstrations; personalized exercise plans; daily movement reminders; wearable integration (steps, HR^f^, and HRV^g^)	AI^h^-driven motion feedback (computer vision); adaptive load progression; safety alerts; and gamification	Physical function (FIQ physical domain), fatigue, adherence (logged sessions), and HR and HRV trends
Mind-body techniques (mindfulness, Tai Chi, breathing, and relaxation)	Moderate evidence for improving sleep, mood, and QoL^i^	Audio-guided meditations, breathing animations, mindfulness timers, and relaxation music	Adaptive session lengths; stress biofeedback using HRV; sleep tracking linkage; and integration with smartwatches	Sleep quality, stress index, anxiety, HRV, and app usage continuity
Symptom tracking and patient-reported outcomes	Essential for personalization and clinical insight for HCP^j^	In-app FIQ, pain diaries, and fatigue and sleep trackers	Voice or video symptom entry; AI-based summary visualization; and adaptive dashboard	Data completeness, trend accuracy, and correlation with clinical outcomes
Personalization and adaptive design	Increasingly essential for engagement and relevance	Custom goal setting; phenotype-based pathways (eg, obesity-, menopause-, and PTSD^k^-related FM^l^)	Machine learning–based tailoring; predictive suggestions for pacing and exercise. Adapted avatars, eg, older people with obesity	User satisfaction, engagement over time, and adaptive accuracy
Behavior change and motivation	Crucial for long-term adherence	SMART^m^ goal planning, feedback loops, and progress visualization	Gamification, motivational messaging, positive reinforcement, and social comparison (optional)	Retention rate, adherence index, and self-efficacy gain
Communication and support	Improves adherence and patient safety	Chatbot or professional chat; asynchronous therapist feedback	Hybrid care integration (eg, AI triage + human follow-up); crisis escalation paths	Message frequency, satisfaction, and safety events
Data integration and clinical workflow	Enables clinical supervision and research	Clinician dashboard, FHIR^n^-based interoperability, and data export to EHR^o^	Sidecar EMR integration; secure teleconsultation channel	Clinical uptake, data completeness, and clinician feedback
Accessibility, UX^p^, and emotional design	Critical for usability and adherence in cognitive fatigue	Clean interface, large icons, voice navigation, and light and dark modes	Emotionally supportive design (colors, feedback tone), simplified onboarding, and language localization	SUS^q^ score, accessibility compliance, and dropout rate
Privacy, certification, and ethics	Required for trust and scalability	CE^r^, FDA^s^, or DiGA^t^ conformity, and transparent data policies	Privacy-by-design architecture; on-device data processing	Certification status, GDPR^u^ or HIPAA^v^ compliance, and user trust rating

^a^CBT: cognitive behavioral therapy.

^b^ACT: acceptance and commitment therapy.

^c^PNE: pain neuroscience education.

^d^RCT: randomized controlled trial.

^e^FIQ: Fibromyalgia Impact Questionnaire.

^f^HR: heart rate.

^g^HRV: heart rate variability.

^h^AI: artificial intelligence.

^i^QoL: quality of life.

^j^HCP: health care professional.

^k^PTSD: posttraumatic stress disorder.

^l^FM: fibromyalgia.

^m^SMART: specific, measurable, achievable, relevant, time-bound.

^n^FHIR: Fast Healthcare Interoperability Resources.

^o^EHR: electronic health record.

^p^UX: user experience.

^q^SUS: System Usability Scale.

^r^CE: Conformité Européenne.

^s^FDA: US Food and Drug Administration.

^t^DiGA: Digitale Gesundheitsanwendung.

^u^GDPR: General Data Protection Regulation.

^v^HIPAA: Health Insurance Portability and Accountability Act.

**Recommendations for the fibromyalgia mobile health self-management program development.**

**Recommendation**

- Involve key stakeholders (fibromyalgia patients, multidisciplinary professionals, and digital experts) through surveys, interviews, and co-design workshops.
- Conduct a structured needs assessment, addressing core fibromyalgia symptoms and patient priorities (eg, fatigue, pain, and cognitive dysfunction).
- Provide a transparent overview of the content for users, health care providers, and regulators, eg, using knowledge graphs or content maps.
- Tailor content to user characteristics such as socioeconomic background, gender, age, culture, and health literacy.
- Base all interventions on clinical evidence, including physical activity, cognitive behavioral therapy, psychoeducation, and mind-body strategies.
- Include personalized modules for subtypes and comorbidity (eg, perimenopausal symptoms, obesity, anxiety, posttraumatic stress disorder–associated hypermobility, migraine, etc).
- Adhere to established standards and guidelines, such as Xcertia, National Institute for Health and Care Excellence, Haute Autorité de Santé, and World Health Organization, covering content, privacy, usability, and operability.
- Design the app as a complementary tool, not a substitute for face-to-face care.
- Use interoperable data formats, such as Fast Healthcare Interoperability Resources, to enable secure integration with health care systems and devices.
- Connect to wearable data, allowing tracking of physical activity, sleep, or heart rate variability to enrich outcome measures.
- Include a clinician-facing dashboard to enable remote monitoring, triage, and decision support.
- Ensure robust data security and privacy compliance, including user consent, encryption, and data governance.
- Provide a structured and simple onboarding process, guiding users through initial setup and goals.
- Ensure user-friendliness with intuitive design, a responsive user interface, customizable settings, reminders, and accessible language.
- Include motivational features, such as gamification, progress tracking, and positive reinforcement.
- Incorporate core functional modules, including symptom tracking, educational content, communication, and self-assessment tools.
- Implement adaptive, guided interventions rather than static or generic content to boost engagement and outcomes.
- Define and track recognized fibromyalgia end points to support evidence generation and reimbursement (eg, Fibromyalgia Impact Questionnaire, pain scales).
- Follow an iterative development cycle, including continuous evaluation of effectiveness, usability, and safety.
- Plan for scalability, dissemination, and regulatory approval, including Conformité Européenne marking, Digitale Gesundheitsanwendungen eligibility (Germany), or US Food and Drug Administration listing.

## Areas of Uncertainty

The literature search was limited to PubMed and Cochrane databases, potentially introducing selection bias, and the cutoff at 2023 excluded recent studies or emerging interventions. Another limitation of this viewpoint concerns the transferability of conventional evidence-based therapies to digital formats. The effectiveness of individual therapeutic elements within apps remains largely untested, and user experience likely plays a decisive role. Adherence is particularly critical, as unguided online interventions often show low engagement. Psychiatric comorbidities, such as depression, trauma, and cognitive fatigue, further affect adherence and suggest that some users may benefit from guided or coach-assisted programs rather than fully automated ones. The human and group-based components of multimodal therapy are difficult to replicate digitally, raising questions about the optimal degree of human involvement in digital therapeutics. Device-based or biofeedback interventions were not included, as they require additional hardware not universally accessible; however, future versions may integrate smartwatch-based or wearable data.

Finally, personalization remains a key challenge. As fibromyalgia is heterogeneous, tailored approaches—whether based on symptom profiles or machine learning–derived phenotypes—are needed. Future studies should also account for contextual factors such as age, culture, digital literacy, and socioeconomic status, as well as identify which behavior change techniques drive engagement and outcomes. Guided and adaptive interventions appear more effective than static content, highlighting the importance of iterative, patient-centered design in future mHealth development.

## Future Directions

As seen in other digital interventions, such as for depression, the inclusion of a channel for direct interaction with a health care professional or, potentially, a bot can increase effectiveness. Voice message transcription features can facilitate semiautomated, asynchronous communication. In this context, agentic AI trained on such interactions may provide supplementary or alternative support. Dashboards play a critical role in integrating fibromyalgia mHealth applications into the clinical workflow, enabling health care professionals to monitor their patients and app activity. Ideally, these dashboards should interface with electronic medical records through sidecar applications [81]. Personalization of content according to fibromyalgia subtypes should be encouraged. However, this increased individualization may make the creation of robust evidence or standardized certification more challenging. As a future perspective, new content formats could be developed and evaluated, such as therapeutic stories or short interventions. For example, therapeutic stories could resemble YouTube Shorts, with AI algorithms applied to tailor and deliver these formats accordingly. Other future digital directions for fibromyalgia apps will move beyond psychoeducation toward multimodal, personalized therapy ecosystems. Integration of exercise modules, CBT, and mindfulness into cohesive platforms is expected, with adaptive interfaces that adjust content to each user’s symptom patterns, motivation, and cognitive load. LLMs and AI-powered chatbots will evolve from static text-based assistants to emotionally intelligent digital companions that guide users through daily self-management, provide empathetic feedback, and interface with sensor data from wearables for sleep, mobility, or stress tracking. These systems could enhance adherence, detect flares early, and personalize pacing and exercise recommendations. Meanwhile, interoperability, data privacy, and regulatory validation will remain crucial to ensure clinical reliability and ethical use. Ultimately, the future of fibromyalgia apps lies in hybrid digital care, combining automated AI-driven support with human coaching and clinical oversight. By blending evidence-based therapy, personalization, and human empathy, next-generation digital therapeutics may finally bridge the gap between self-management and sustained symptom improvement.

## Conclusion

Evidence-based foundations are needed to inform the development of effective digital therapeutic applications for fibromyalgia. To build impactful digital therapeutics, it is essential to integrate validated self-management strategies, engage key stakeholders, and consider social and cultural determinants. An iterative development process, guided by ongoing assessment of usability, effectiveness, equity, and safety, will ensure that mHealth tools align with both clinical goals and patient needs.

## Appendix

Multimedia Appendix 1 Supplementary tables and figures.

## Abbreviations

ACT: acceptance and commitment therapy
AI: artificial intelligence
CBT: cognitive behavioral therapy
DiGA: Digitale Gesundheitsanwendung
FIQ: Fibromyalgia Impact Questionnaire
LLM: large language model
mHealth: mobile health
MMT: meditative movement therapy
QoL: quality of life

## Data Availability

All data generated or analyzed during this study are included in this published article. Quantitative and qualitative data of focus groups can be found in [78].
