# Supplementary material for: Evidence-Based Self-Management Strategies for Fibromyalgia: Foundations for Digital Therapeutic Applications
Source: Interact J Med Res. 2026 Feb 17;15:e67523. doi: 10.2196/67523 (PMC12957949; doi:10.2196/67523)
Supplement: Multimedia Appendix 1 [file ijmr_v15i1e67523_app1.docx]

| **SUPPLEMENTARY DATA**  **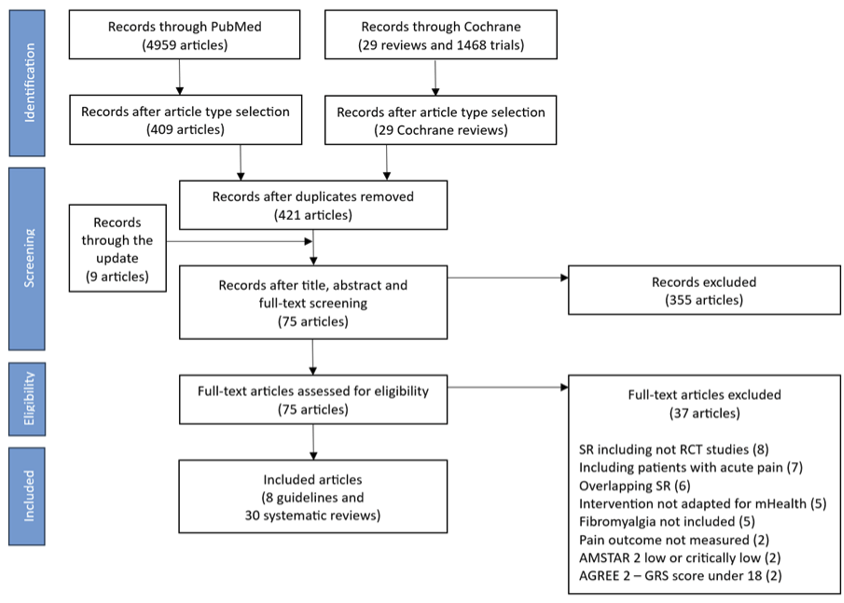**  **Figure S1.** Study selection flowchart of guidelines and systematic reviews on randomized controlled trials. SR: systemic review, RCT Randomized controlled trials.  **Table S1. Interventions characteristics** | | | | | | |
| --- | --- | --- | --- | --- | --- | --- |
|  |  |  |  |  |  |  |
| **Type and sources** | | **Description** | **Frequency and Intensity** | **Time and Time Frame** | **Delivery modalities** | **Safety** |
|  |  |  |  |  |  |  |
| PHYSICAL | AE  [[60,62,65,66]](https://www.zotero.org/google-docs/?75ncuE) | Authors suggested AE should be simple and easy activities.  *Options such as walking, aerobic jogging, ergometer, exergames, cycling, dance, rhythmic movements, and exercise circuits. | Three times a week.  AE should commence at a level slightly below the individual's physical capacity and be progressively escalated. | 30-60 minutes.  A minimum of four weeks with the most significant pain reduction occurs between 12 and 24 weeks.  As recommended by WHO, by embracing a physically active lifestyle, individuals have the potential to sustain the benefits acquired. | The choice between group or individual sessions should be based on the patient's preference, considering that depression may be a limiting factor for group sessions.  Land-based AE are recommended, but aqua-based aerobic exercises can provide additional benefits due to the properties of water. However, accessibility and cost should be taken into consideration. | AE are well tolerated when individuals adhere to instructions that help prevent symptom exacerbation. However, high-intensity exercises can result in non-adherence to training. The risk of adverse effects associated with AE was found to be low. |
|  | RE  [[60,63,65,66]](https://www.zotero.org/google-docs/?Bp8zwh) | *RE for the lower and upper limbs and the rachis.  *With resistance machine, free weights, or body weight for resistance. | Two to three times a week  Begin RE with a low intensity, below the individual's physical capacity. The intensity should then be progressively increased from low to moderate.  The exercise regimen should be adapted based on the patient's perception and feedback. | 30-60 minutes.  1-2 sets with 4-20 repetitions.  There was a significant impact on pain reduction during the periods of 8-12 weeks and 15-24 weeks. | Land-based RE are recommended, but aqua-based RE can provide additional benefits due to the virtues of water.  However, it is important to consider factors such as accessibility and cost.  Group supervision were suggested to improve motivation. | Instruction should be given to prevent symptom exacerbation.  High-intensity RE should be not pursued for prolonged periods. |
|  | FE  [[65,67]](https://www.zotero.org/google-docs/?VuNXkT) | FE on free pain muscles. | 1-3 times a week progressively increased up to five times a week.  The stretching should be performed until the point of tightness or a mild level of discomfort is reached. | The duration of FE should initially be maintained between ten and 30 seconds, gradually progressing up to 60 seconds.  *2-4 repetitions during a session of 40-60 minutes.  (no information about the time frame) | (no information) | Well tolerated with low quality of evidence. |
|  |  |  |  |  |  |  |
| PSYCHO-  EDUCATIVE | CBT [[14,28,68,70-72,76,84]](https://www.zotero.org/google-docs/?7Mlhay) | *CBT for pain: education of pain, relaxation, emotion identification, planning activities, assertive communication training, problem solving, pain-specific cognitive therapy, pain catastrophising and safety-seeking behaviour reduction, reversing mental defeat, integration of treatment component, pacing , relapse prevention, goal setting, self-management education, operant therapy, ACT, MBT.  *CBT for insomnia: education of sleep, sleep schedule restriction, problem solving, stimulus control, cognitive therapy?, relaxation, activity-rest cycle, visual imagery, review of skills and maintenance, relapse prevention. | *Weekly, on demand, or at the set time point | *18 (3-102) hours on 10 (6 to 60) sessions  Authors observed a stronger effect for treatments with longer durations. Treatments lasting 10 weeks, with a range of 5 to 54 weeks, yielded positive outcomes. | CBT were typically conducted in a face-to-face format, but they could also be delivered through internet-based or telephone methods.  These interventions can be provided in either a group or individual format.  Regarding internet-based CBT, there are two main approaches: guided and unguided. In guided internet CBT, materials are provided through the internet, and individuals receive support and guidance via email, messages, or telephone calls. The impact of therapist qualifications and experience on guided internet CBT interventions remains unclear. On the other hand, both guided and unguided internet CBT interventions have shown improvements in depression, self-efficacy, and pain catastrophizing. However, guided interventions have demonstrated superior effects on disability, anxiety, and pain intensity.  Internet-delivered ACT were as effective as face-to-face formats in addressing chronic pain.  Various forms of material were utilized in the interventions, including written materials, emails, videos, audios, text messages, illustrations, telephone consultations, metaphors, and pain diaries. | CBT interventions were associated with a low risk of adverse effects. Subjects showed high levels of adherence to ACT. |
|  | Education  [[14,73-75,77]](https://www.zotero.org/google-docs/?7nVH0F) | Fibromyalgia disease and its management explanation, the reconceptualization of misconceptions and myths.  TPNE including the neurophysiology of pain, the nervous system plasticity, the psychosocial factors and beliefs.  Education on everyday life management, the self-management approach, physical activities, CBT, the pharmacological treatment guidance.  About physical activities, CBT, general life. | The effectiveness of TPNE was not found to be influenced by the frequency. | The effectiveness of TPNE was not found to be influenced by the duration. | The effectiveness of TPNE was not found to be influenced by the format (whether delivered in a group or individual-based setting), with the exception of fear of movement outcomes, which demonstrated better results when the intervention was provided in a group format.  *Discussion group, brochures and informative books, talks and informative lectures, visual information  *The book Explain Pain by Moseley and Butler (2003) was repeatedly used  *By professionals (such as physical therapists, occupational therapists), face-to-face, individual or in group, or self-guided, tailored or not. | (no information) |
|  |  |  |  |  |  |  |
| MIND-BODY | Meditative movement therapy  [[80,82,84]](https://www.zotero.org/google-docs/?wDNx8r) | Tai Chi, Yoga, Qi Gong  *There are different Tai Chi styles are practised. Although that the Yang style is the most popular, all styles appeared efficient. | (no information) | Despite the lack of evidence, longer duration (24 weeks) showed better results than short duration of Tai Chi.  (no information about the duration) | (no information) | While MMTs are generally considered relatively safe, precautions should still be taken due to the potential risk of increasing pain or musculoskeletal injuries.  It is important to note that a large number of participants dropped out of the study due to organizational reasons and fatigue induced by attending the course. |
|  | Meditation  [[83]](https://www.zotero.org/google-docs/?tQErZs) | *Mindfulness-based stress reduction, which is also utilized as a CBT technique, incorporates various meditation practices such as sitting and walking meditation, body scan, Hatha yoga, and loving-kindness meditation. | (no information) | *45-150 minutes.  *Over 8-12 weeks. | *Training guided session continued by self-programs.  *All day retreat were proposed.  *Usually in group format. | No adverse effect reported |
|  | Relaxation  [[83]](https://www.zotero.org/google-docs/?IxUIUu) | *Progressive muscle relaxation, breath therapy. | (no information) | *8-10 hours over 2-10 weeks. | Therapist assisted and self-guided were equally effective. | Relatively safe with one adverse effect reported (uncomfortable memories).  These interventions were considered relatively safe in their study. |
|  | Hypnosis, guided imagery and autogenic training  [[83]](https://www.zotero.org/google-docs/?CebIhm) | (no information) | (no information) | *21-47 hours over three days to eight weeks. | *Face-to-face or self-administrated with audios or auto-suggestions. | A single adverse effect, namely mild transient headaches, was reported, and it should be noted that these practices may exacerbate symptoms in individuals with psychiatric or heart conditions, epilepsy, a history of abuse, or trauma. |
|  |  |  |  |  |  |  |
| *The following suggestions are based on the average information available, but no specific recommendation is given.  AE= Aerobic Exercices; WHO= World Health Organization; RE= Resistance Exercises; FE= Flexibility Exercises; CBT= Cognitive-Behavioural Therapy; ACT= Acceptance and Commitment Therapy; MBT= Mind-Body Therapy; TPNE= Therapeutic Pain Neuroscience Education; MMT= Meditative Movement Therapy. | | | | | | |
